# Augmented vs. standard glenoid baseplate use in reverse total shoulder arthroplasty: a systematic review

**DOI:** 10.1016/j.jseint.2025.05.032

**Published:** 2025-06-14

**Authors:** Emily N. Lau, Ryan Lin, Abbey Glover, Albert Lin

**Affiliations:** a Department of Orthopaedic Surgery, University of Pittsburgh Medical Center, Pittsburgh, PA, USA; b University of Pittsburgh School of Medicine, Pittsburgh, PA, USA; c Drexel University College of Medicine, Philadelphia, PA, USA

**Keywords:** Reverse total shoulder arthroplasty, Glenoid baseplate, Augmentation, Glenoid bone loss, Glenoid wear, Glenoid preservation

## Abstract

**Background:**

Glenoid bone loss can be a challenging problem to address surgically in patients with glenohumeral joint arthritis and concomitant end-stage rotator cuff deficiency. Reverse total shoulder arthroplasty (rTSA) has emerged as a treatment to restore function in this patient population and has demonstrated good clinical outcomes. Addressing bone loss is essential to prevent complication such as glenoid baseplate loosening, scapular notching, and instability. Metal augmentation of glenoid baseplates has shown good clinical and functional outcomes with low complication rates. This systematic review analyzes the outcomes of patients undergoing rTSA with augmented baseplates vs. those treated with a standard glenoid baseplate. Pain, range of motion, patient reported outcomes scores, complication, and revision rates were assessed.

**Methods:**

Four online literature databases (PubMed, MEDLINE, ScienceDirect, Scopus) were searched from database inception to July 1, 2024, for comparative studies evaluating outcomes between augmented and standard rTSA. Functional and clinical outcomes along with complication and revision rates were collected across studies. Frequency weighted means were used to synthesize data where appropriate.

**Results:**

Five manuscripts met final criteria for inclusion encompassing 2,331patients with a mean age of 71.3 years and mean follow-up time of 38.8 months. When compared to the standard baseplate group or those treated with bone graft augmentation, the metal augmentation group had comparable improvement in frequency weight means in forward elevation, abduction, and external rotation. Similarly, frequency weighted means of improvement were comprable in the augmented group with regards to American Shoulder and Elbow Surgeons, Simple Shoulder Test, and Constant scores. In studies directly comparing augmented to nonaugmented rTSA, there was a total of 167 (7.5%) reported complications: 132 (7.8%) in the standard and 35 (6.7%) in the augmented cohorts.

**Conclusion:**

This systematic review demonstrates similar functional and clinical outcomes with the use of augmented glenoid baseplates to address glenoid bone loss in rTSA when compared to standard baseplates. Complications were comparable in the augmented baseplate group, with no difference in revision rates. These findings illustrate that augmented baseplates not only address bony defects but can provide good clinical and functional outcomes without the risk of increased complication in rTSA.

Glenohumeral joint arthritis affects the majority of men and women over 80 years of age.^20^ In patients with concomitant end-stage rotator cuff deficiency, superior humeral head migration and subsequent glenoid wear, most often posterior, are not uncommon.^6^^,^^15^ Reverse total shoulder arthroplasty (rTSA) has become more common as the average age of individuals continues to rise, and treatment with rTSA has been shown to successfully restore function in this patient population. Although rTSA has demonstrated good clinical outcomes, glenoid wear is a challenging problem, and addressing bone loss is critical to prevent complications such as glenoid baseplate loosening, scapular notching, and instability.^13^

Several techniques have emerged to address glenoid wear. Eccentric reaming is a simple and low-cost technique that involves asymmetric reaming of the glenoid to produce a uniform surface for baseplate implantation.^1^ This technique has been shown to be effective in addressing smaller bony defects; however, larger areas of bone loss demand an alternative treatment approach to avoid massive amounts of bone removal. Bone grafting has also been trialed in efforts to mitigate bone loss from reaming. However, some studies have shown high risk of failed incorporation of bone graft and resorption, with use of both allograft and autograft, leading to poor outcomes. This technique is also more technically demanding.^12^^,^^19^^,^^21^

More recently, metal augments have been added to glenoid base plates to address bony defects.^4^^,^^5^^,^^7^^,^^10^^,^^11^^,^^21^, ^22^, ^23^ In theory, these augments can fill larger defects in the glenoid without the risk of over reaming and subsequent bone loss. Furthermore, the risk of nonincorporation or resorption is mitigated.^14^ Augmented base plates have shown positive clinical outcomes in addressing glenoid wear, reducing pain, increasing range of motion (ROM) and improving patient reported outcomes and functional scores.^2^ Good radiographic outcomes are well documented along with low complication and revision rates.^2^^,^^7^ Figure 1 demonstrates preoperative and postoperative radiographs of a patient who underwent a nonaugmented rTSA, while Figure 2 demonstrates radiographs for a patient whom underwent an augmented construct. Despite several studies report positive outcomes after augmented rTSA, there is a lack of comparative analyses of augmented vs. nonaugmented rTSA across the literature. As such, the objective of this systematic review is to analyze clinical outcomes of rTSA with metal augmented baseplates vs. standard constructs. We hypothesized that clinical outcomes and complication rates would be similar in the augmented cohort compared to the standard cohort.

**Figure 1:**
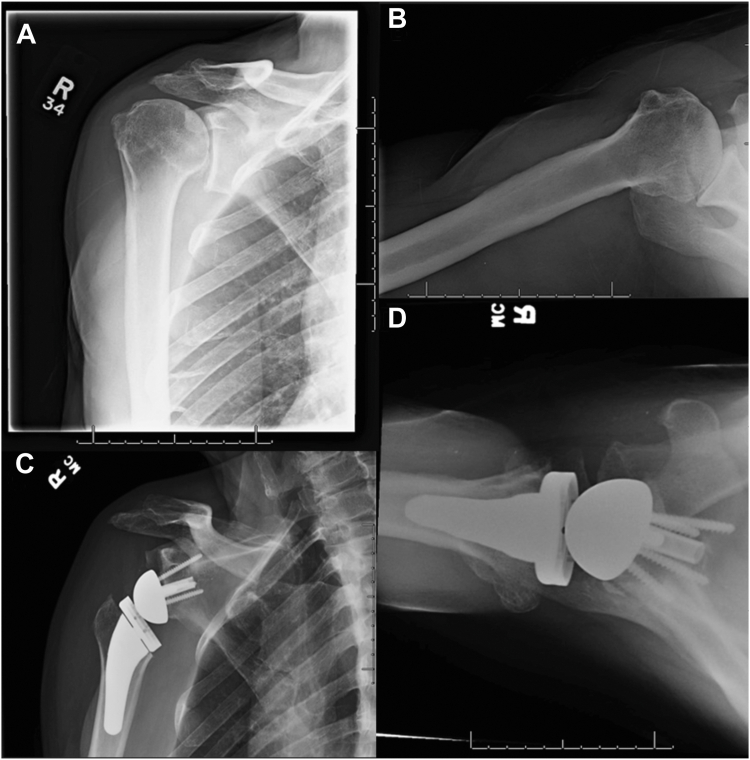
Preoperative anteroposterior (**A**) and axillary (**B**) views and postoperative anteroposterior (**C**) and axillary (**D**) views of nonaugmented rTSA. *rTSA*, reverse total shoulder arthroplasty.

**Figure 2:**
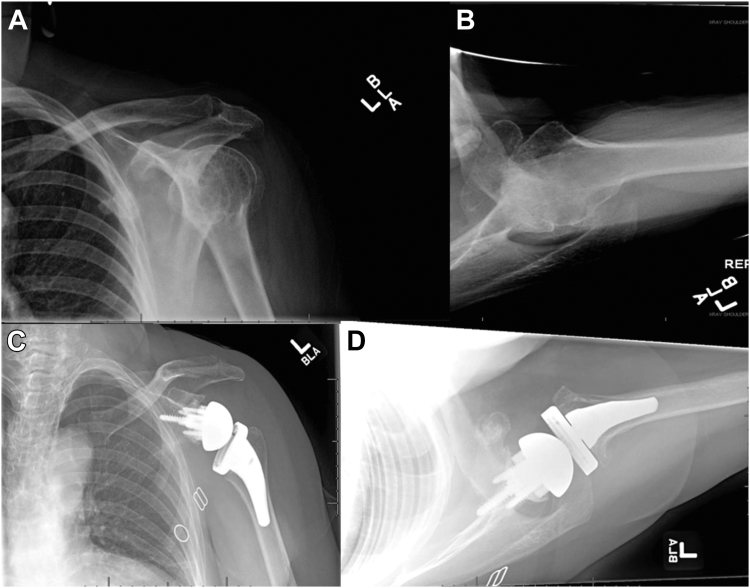
Preoperative anteroposterior (**A**) and axillary (**B**) views and postoperative anteroposterior (**C**) and axillary (**D**) views of augmented rTSA. *rTSA*, reverse total shoulder arthroplasty.

## Materials and methods

**Figure 3:**
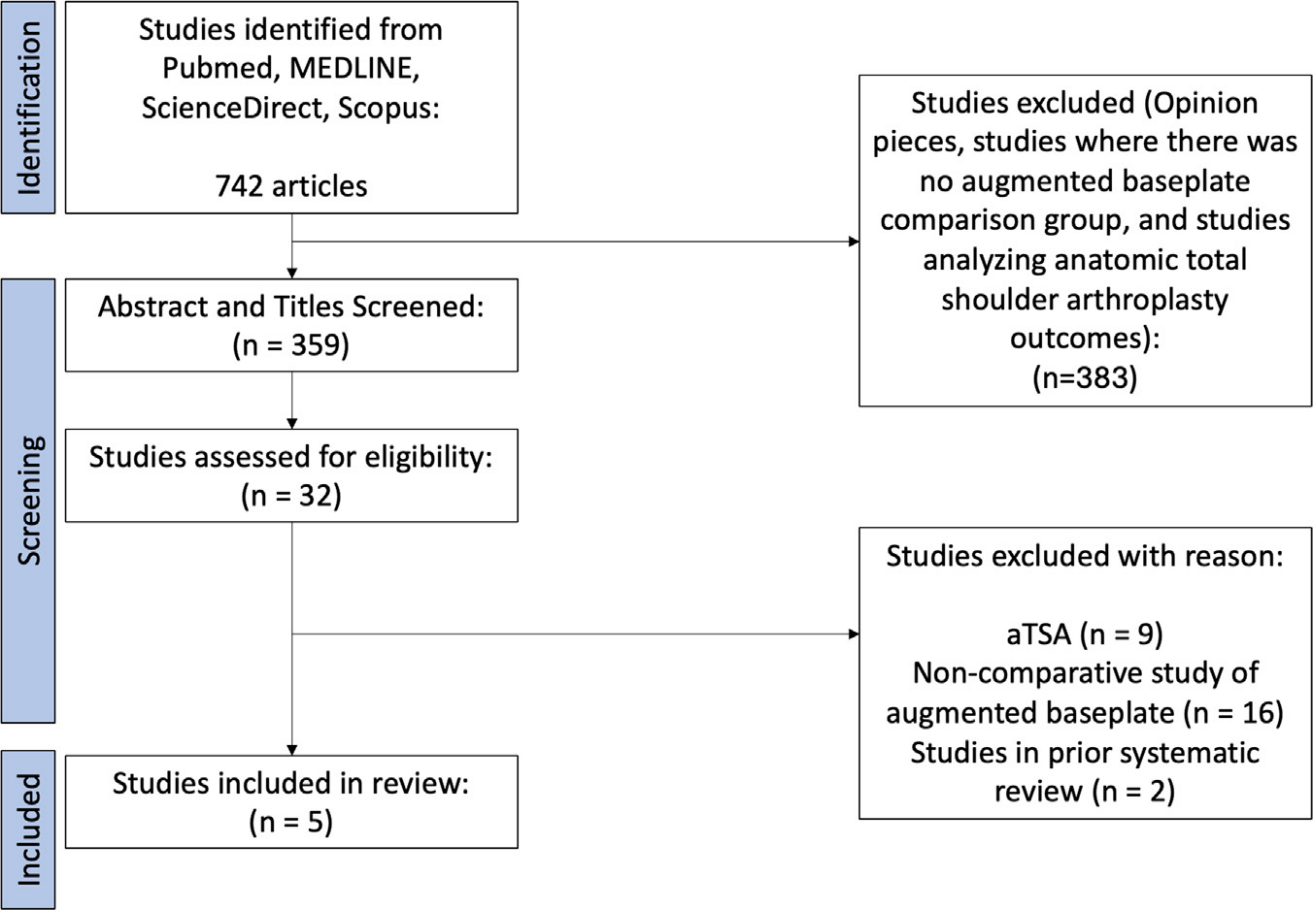
PRISMA flow diagram for study selection and inclusion. *PRISMA*, Preferred Reporting Items for Systematic Reviews and Meta-Analyses; *aTSA*, anatomic total shoulder arthroplasty.

The systematic review was conducted using the Preferred Reporting Items for Systematic Reviews and Meta-Analyses guidelines (Fig. 3).

### Search strategy

Four online literature databases (Pubmed, MEDLINE, ScienceDirect, Scopus) were searched from database inception to July 1, 2024, for comparative studies evaluating outcomes between augmented and standard rTSA. Search terms including reverse shoulder arthroplasty, RSA, rTSA, augment, augmented baseplate, standard baseplate, and glenoid were utilized.

### Inclusion and exclusion criteria

Inclusion and exclusion criteria were determined a priori. Inclusion criteria were defined as studies (1) involving patients 18 years or older, (2) undergoing primary or revision rTSA, (3) comparing patient cohorts where at least 1 cohort received an augmented glenoid baseplate, and (4) English-language. Opinion pieces, studies where there was no augmented baseplate comparison group, and studies analyzing anatomic total shoulder arthroplasty outcomes were excluded.

### Study evaluation and selection for incorporation

Screening and data extraction of articles was performed independently by 2 authors (E.L. and A.G.). In reviewing articles for incorporation in this review, any article with controversy regarding inclusion was discussed and a consensus was reached between the 2 reviewers. Reference lists of all included studies were assessed for any additional studies meeting criteria for inclusion.

### Outcomes collection

Outcomes were collected independently by 2 authors (E.L. and A.G.) and discussed thereafter to ensure proper interpretation and reporting in this review. Data were compiled manually by the research team. Outcomes of interest that were identified and compared across studies were as follows: functional scores, pain scores, ROM, revision rates, and complication rates. Frequency weighted means were calculated for the outcomes that were reported in at least 3 studies, and mean clinically important differences were subsequently calculated in standard fashion using JASP Team (JASP version 0.19 2024; University of Amsterdam, Amsterdam, Netherlands).

## Results

### Search

The initial search yielded 742 articles. After preliminary screening by 2 authors independently (E.L. and A.G.), 32 articles remained for full-text review, and subsequently 5 manuscripts met inclusion criteria for final analysis. All manuscripts were Level III comparative studies, in which at least 1 comparison group received metal augmentation of the glenoid baseplate. The mean number of patients in each study was 466 (range 16-1961), with a total of 2,331 pooled patients. In studies with available data, the mean age of patients was 71.3 years. The average follow-up time was 38.8 months. A summary of study characteristics can be found in Table I.

**Table I:** Summary of included studies.

Study	Study design	Mean age, yr	Mean FU mo	Augment characteristics	Primary vs. revision surgery	Implants used	Surgical indications	Clinical outcomes					Functional outcomes								Complications	Revisions
								Forward flexion	Abduction	Passive ER	Active ER	IR	ASES	SAS	SST	GSF	SPADI	UCLA	Constant	VAS		
Levin et al^10^	Level III; Retrospective Cohort Comparison; Treatment Study	SBP: 70.0 ± 8.7 ABP: 73.3 ± 7.7	__________	84 SBP 14 SAB 47 PAB 26 P/SAB	Primary	Exactech	84 SBP (29.8% OA, 42.9% RCT, 67.9% CTA) 87 ABP (59.8% OA, 67.8% RCT, 37.9% CTA)	Y	Y	N	Y	Y	Y	Y	N	Y	N	N	Y	Y	12 (14.3%) SBP 7 (8.0%) ABP	5 (6%) SBP 1 (1.1%) ABP
Gulotta et al^3^	Level III; Retrospective Comparison Study	SBP: 72.0 ± 7.7 PAB: 72.7 ± 7.7 SAB: 74.1 ± 8.6 P/SAB: 69.8 ± 8.3	47.1 ± 23.1	1547 SBP 83 SAB 190 PAB 141 P/SAB	Primary	Exactech	SBP (50.0% OA, 42.3% RCT, 42.1% CTA) SAB (50.6% OA, 37.3% RCT, 57.8% CTA) PAB (77.4% OA, 42.1% RCT, 22.6% CTA) P/SAB (61.7% OA, 36.9% RCT, 29.1% CTA)	Y	Y	N	Y	Y	Y	N	Y	N	Y	Y	Y	N	94 (6.1%) SBP 3 (3.7%) PAB 7 (3.6%) SAB 4 (2.8%) P/SAB	39 (2.5%) SBP 1 (0.5%) PAB 5 (2.4%) SAB 0 (0%) P/SAB
Park et al^18^	Level III; Retrospective Comparison Study (?)	SBP: 72.21 ± 6.06 SAB: 72.13 ± 6.51	SBP: 45.11 ± 16.71 SAB: 43.03 ± 11.54	72 SBP 24 SAB	Primary	Exactech	SBP (18.1% OA, 29.2% RCT, 52.8% CTA) SAB (12.5% OA, 25.0% RCT, 62.5% CTA)	Y	Y	N	Y	Y	Y	N	Y	N	N	N	Y	Y	24 (23.5%) SBP 9 (37.5%) SAB	2 (2.8%) SBP 1 (4.2%) SAB
Krupp et al^8^	Level III; Retrospective Comparison Study (?)	MABP/SHT: 69.5 ± 9 SBP/MHT:68.0 ± 7 MAB/MHT:70.7 ± 3	__________	21 MABP/SHT 23 SBP/MHT 23 MAB/MHT	Primary	Zimmer Biomet	MABP/SHT (23.8% OA, 9.5% RCT, 61.9% CTA) SBP/MHT (8.7% OA, 21.7% RCT, 65.2% CTA) MAB/MHT (30.4% OA, 13.0% RCT, 56.5% CTA)	Y	N	N	Y	Y	Y	N	N	N	N	N	N	Y	6 (28.6%) MABP/SHT 3 (13.0%) SBP/MHT 9 (39%) MAB/MHT	0
Nabergoj et al^16^	Level III; Retrospective Cohort Comparison; Treatment Study (?)	BMA: 72.1 ± 11.7 BA: 73.3 ± 6.8	BMA: 28.1 ± 15.0 BA: 30.7 ± 10.8	8 BMA 8 BA	Primary and Revision	Stryker	BMA (62.5% OA, 12.5% PTA, 12.5% DA, 12.5% Revision) BA (100.0% OA)	Y	N	N	Y	Y	Y	N	N	N	N	N	Y	N	∗	_________

*FU*, follow-up; *ER*, external rotation; *IR*, internal rotation; *ASES*, American shoulder and Elbow Surgeons; *SST*, simple shoulder test; *SPADI*, shoulder pain and disability index; *VAS*, visual analog pain score; *SAS*, shoulder arthoplasty smart; *GSF*, global shoulder function; *SBP*, standard baseplate; *ABP*, augmented baseplate; *SAB*, superiorly augmented baseplate; *P/SAB*, posterior/superior augmented baseplate; *PAB*, posteriorly augmented baseplate; *MAGB*, mini-augmented glenoid baseplate; *STH*, standard humeral tray; *MHT*, mini-humeral tray; *VRS*, vault reconstruction system; *BG*, bone graft; *BMA*, bony-metallic augmentation; *BA*, bony augmentation; *L-RSA*, lateralized reverse shoulder arthroplasty^17^; *S-RSA*, original Grammont-style reverse shoulder arthroplasty; *OA*, osteoarthritis; *RCT*, rotator cuff tear; *CTA*, cuff tear arthropathy; *PTA*, post-traumatic arthritis; *DA*, dislocation arthropathy.

∗ No data available.

### Study characteristics

Three retrospective comparative studies evaluating clinical and functional outcomes, complications, and revision rates between augmented and standard baseplates in rTSA were analyzed. This included 2,228 total rTSA cases: 1,703 with standard and 525 with augmented baseplates. Patients were evaluated at a minimum 2 year follow-up across multiple hospital networks.^3^^,^^10^^,^^18^ One additional study by Krupp et al comparing outcomes of variations of the augmented glenoid baseplate was also reviewed. This study compared the following constructs: group 1 with 21 patients with a mini-augmented glenoid baseplate and standard humeral tray, group 2 with 23 patients with a standard baseplate and mini humeral tray, and group 3 with 23 patients with a mini augmented glenoid baseplate and mini humeral tray.^8^ Augmentation type varied between studies and was as follows: 4 studies utilized metal backed augmented glenoid components, while 1 study did not specifically report on augment composition.

Indications for rTSA across studies were as follows: Levin et al included patients with severe glenoid wear plus rotator cuff tear (RCT) or a cuff tear arthropathy (CTA) diagnosis. Of the patients in the standard group in this study, 29.8% had osteoarthritis (OA), 42.9% had a RCT and 67.9% CTA, while the augmented group had demonstrated 59.8% with OA, 67.8% with RCT and 37.9% with CTA. In a study by Gulotta et al, the indication for rTSA was glenoid bone loss. The superior augment group was more likely to have CTA (*P* = .0047) compared to the standard group. The posterior-superior augment group was more likely to have been diagnosed with OA (*P* = .0256) and rheumatoid arthritis (*P* = .0042) than the standard group. Lastly, the posterior augment group was more likely to have been diagnosed with OA (*P* < .0001) or have a CTA diagnosis (*P* < .0001), than the average standard cohort patient. Park et al reported 62.5% of patients had CTA and 25% had large-massive cuff tears in their augmented group, while in the standard group, 52.8% of patients had CTA and 29.2% had large-massive cuff tears.

### Preoperative pain and function

Preoperatively, Gulotta et al found significantly higher Simple Shoulder Test (SST) scores in the augmented cohort compared to the standard cohort, while Levin and Park et al found no significant differences in preoperative functional or pain scores between cohorts.^3^^,^^10^^,^^18^ Levin et al did note that their standard baseplate cohort had significantly greater preoperative forward elevation and internal rotation compared to the augmented baseplate cohort. Krupp et al found no preoperative differences in ROM, EQ-5D-5 L (a standardized questionnaire used to measure health-related quality of life) quality of life, Visual Analog Pain Score (VAS) shoulder pain, and VAS shoulder instability metrics between their cohorts.^8^

### Postoperative range of motion

Both Gulotta et al and Levin et al found postoperative improvements in ROM as measured by active abduction, forward elevation, internal rotation, and external rotation regardless of implant type.^3^^,^^10^ Gulotta et al specifically compared superiorly augmented baseplate, posteriorly augmented baseplate and posterior/superior augmented baseplate with the standard design. Compared to the standard baseplate, the posteriorly augmented baseplate group had significant improvement in all motions except internal rotation, and posterior/superior augmented baseplate group had significant improvement in all motions except forward elevation. The superiorly augmented baseplate group demonstrated significant improvement in external rotation.^3^ Levin et al found significantly greater improvements in ROM and magnitude of postoperative ROM in the augmented baseplate cohort compared with the baseplate cohort at several follow-up time points. Park et al found no difference in the proportion of patients exceeding the minimal clinically important difference (MCID) for ROM between groups. The augmented group showed significant improvement in active forward elevation and abduction postoperatively and the standard cohort was found to have decreased internal and external rotation after surgery.^18^ Krupp et al found the standard baseplate with mini humeral tray (Group 2) and mini augmented glenoid baseplate with mini humeral tray (Group 3) to have significantly greater flexion than the cohort with mini-augmented glenoid with standard humeral tray (Group 1). Group 3 also showed significantly greater flexion vs. Group 2 and significantly greater external rotation at 90 degrees of abduction along with significantly greater internal rotation compared to Groups 1 and 2.^8^

**Figure 4:**
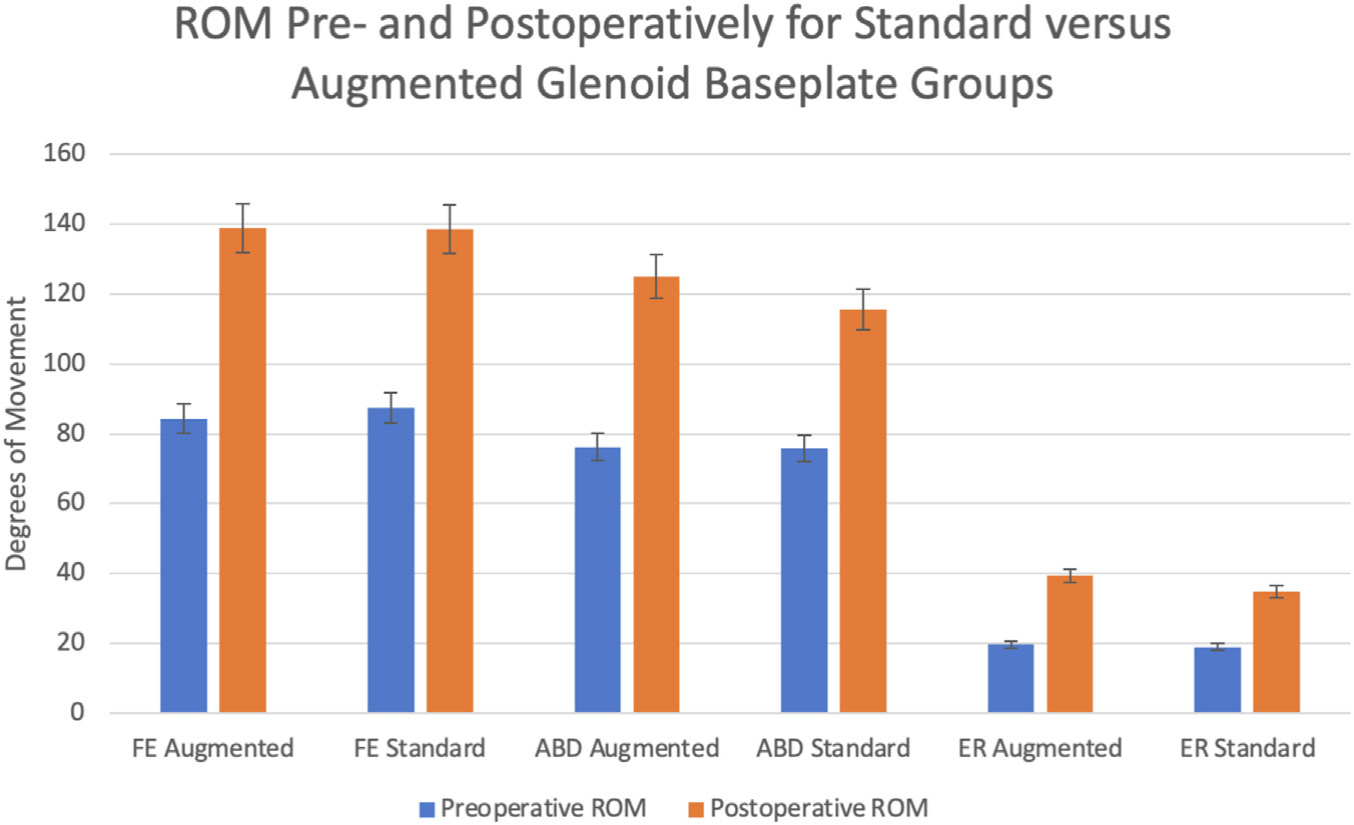
Preoperative and postoperative ROM in augmented and standard rTSA FE (forward elevation), ABD (abduction), ER (external rotation). Degree measurements are frequency weighted means of the ROM reported across the 3 comparative cohort studies. *ROM*, range of motion; *rTSA*, reverse total shoulder arthroplasty.

Frequency-weighted means were calculated for forward elevation, abduction, and external rotation measurements among the 2,228 total patients. Forward elevation increased from 87.3 to 138.4 and 84.3 to 138.7° in the augmented and standard groups respectively. Abduction increased from 76.2 to 124.9 and 75.8 to 115.5°. External rotation increased from 19.7 to 39.4 and 19.0 and 34.9° (Fig. 4). Internal rotation was measured by vertebral segments and quantified using point systems in accordance with vertebral level reached. Gulotta et al reported a significant increase in internal rotation score with the posteriorly, superiorly augmented group specifically compared to the standard group.

### Postoperative clinical outcomes

**Figure 5:**
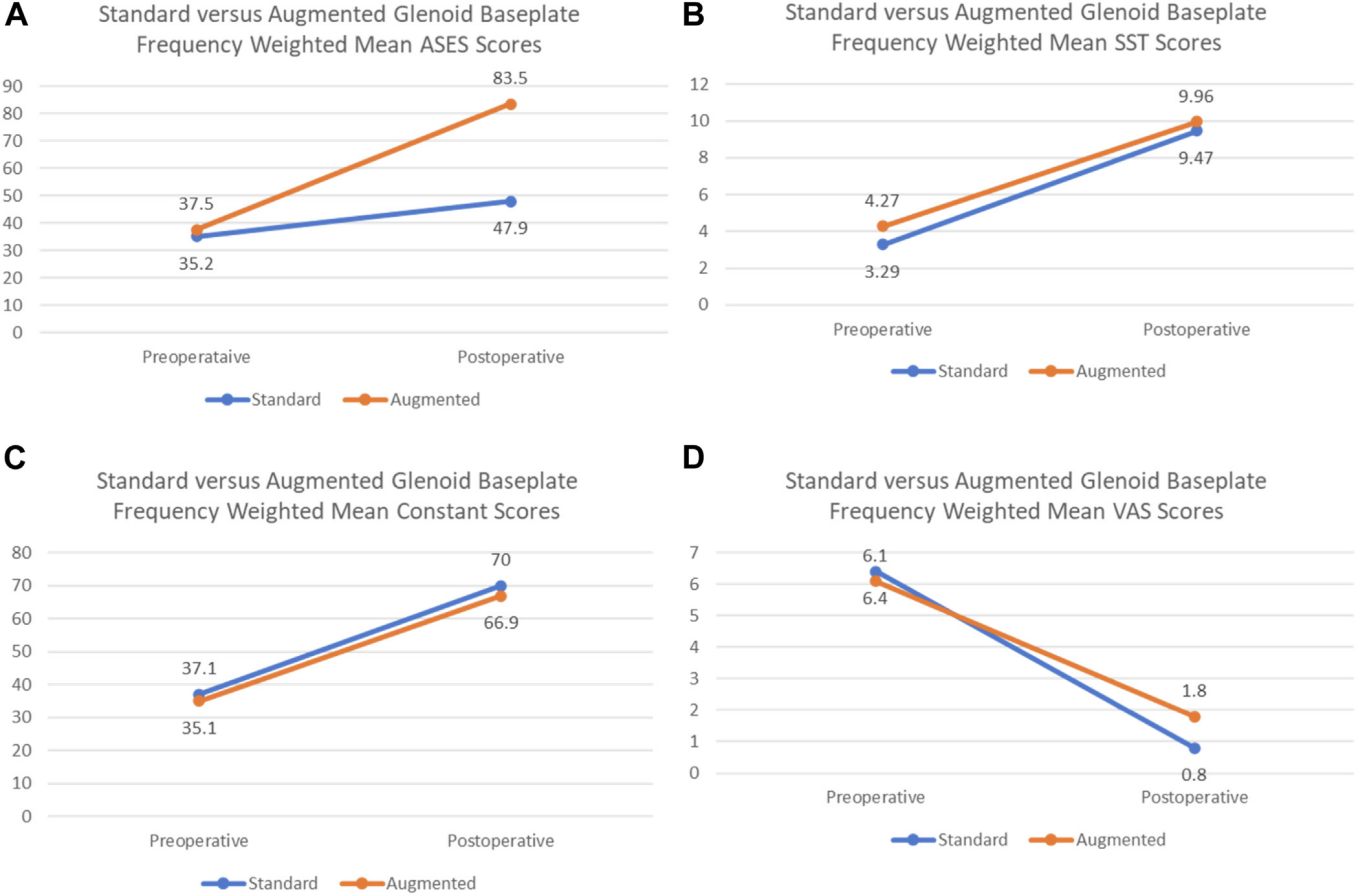
Clinical outcome metrics between standard and metal augmented rTSA between preoperative and postoperative time points (**A**) American Shoulder and Elbow Surgeons score, (**B**) SST sore, (**C**) Constant score, (**D**) VAS. *rTSA*, reverse total shoulder arthroplasty; *VAS*, visual analog scale pain score; *SST*, simple shoulder test; *ASES*, American Shoulder and Elbow Surgeons.

The American Shoulder and Elbow Surgeons (ASES) score was assessed in the Levin, Gulotta and Park studies. The frequency-weighted ASES mean of the 2,228 patients improved from 37.5 to 83.5 and 35.2 to 47.9 in the augmented and nonaugmented groups respectively. The frequency-weighted mean of patients who exceeded the MCID was 93.2% for augmented cohort patients and 91.8% for nonaugmented patients (Fig. 5, *A*). The SST score was assessed in 2 studies. The frequency-weighted mean of these 2057 patients improved from 4.2 to 10.0 and 3.3 to 9.5 in the augmented and nonaugmented groups respectively. The frequency-weighted mean of patients who exceeded the MCID was 87.0% for the augmented group and 90.0% for nonaugmented patients (Fig. 5, *B*). The Constant score was assessed in 3 studies. The frequency-weighted mean of these 2,228 patients improved from 37.1 to 70.0 and 35.1 to 66.9 in the augmented and nonaugmented groups respectively. The frequency-weighted mean of patients who exceeded the MCID was 92.7% for augmented cohort patients and 96.1% for nonaugmented patients (Fig. 5, *C*). VAS score was assessed in 2 of the comparative studies. The frequency-weighted mean of these 2,057 patients improved from 6.4 to 0.8 and 6.1 to 1.8 in the augmented and nonaugmented groups respectively (Fig. 5, *D*).^3^^,^^10^^,^^18^ Krupp et al found the mini augmented glenoid baseplate with mini humeral tray group to have significantly greater postoperative ASES scores at 1 and 2 years follow-up compared to mini-augmented glenoid with standard humeral tray group. However, VAS shoulder pain and instability scores, EQ-5D-5 L scores, and shoulder strength were not significantly different between the 3 cohorts postoperatively.^8^

### Glenoid augmentation vs. bone graft

There have been several studies published on the clinical outcomes of bone graft augmentation in rTSA and subsequent comparison to metal augments. Because this systematic review focuses on augmented vs. standard glenoid baseplate outcomes in rTSA, only a brief summary of comparative outcomes between metal and bone graft augmentation is included. In the systematic review of 19 studies comparing rTSA with supplemental bone graft or baseplate augmentation by Lanham et al, similar overall complication rates, revision rates, ROM, and functional outcome scores were found between groups. Infection, component loosening, and notching were more common in the bone graft group.^9^ Nabergoj et al demonstrated significant postoperative improvements in ROM and clinical scores after 2 years in rTSA with bony-metallic augmentation (BMA) and rTSA with bony augmentation (BA) alone.^16^ The BMA group had greater anterior forward flexion and Constant score. Comparing augmented rTSA to rTSA with bone graft supplementation, Jones et al reported significant improvements in ROM and clinical metric scores in both groups. However, the bone graft cohort had greater rate of scapular notching and overall complication rate.^4^

### Complications

Among the 3 comparative studies of augmented vs. non augmented constructs with 2,228 pooled patients, there were 167 (7.5%) reported complications: 132 (7.8%) in the standard and 35 (6.7%) in the augmented cohorts.^3^^,^^10^^,^^18^ Park et al found a significantly lower complication rate in the augmented cohort, which had 9 complications: 4 scapular notching, 3 acromial fractures, 1 dislocation, and 1 periprosthetic fracture. The standard baseplate cohort had 26 complications in this study: 24 scapular notching and 2 periprosthetic fractures (*P* = .008).^18^ Levin et al reported a trend toward significantly higher revision rate in the standard baseplate cohort compared to augmented cohort: 6.0% and 1.1% respectively (*P* = .088). Neither of the aforementioned studies demonstrated a significant difference in reoperation rate between cohorts. Revision indications reported by Levin et al included periprosthetic fracture (5 standard), glenoid baseplate loosening (2 standard), infection (1 augmented, 1 standard) and humerus fracture (1 standard).^10^ Gulotta et al found no significant difference in complication, nor revision rates between cohorts.^3^ Krupp et al also found no statistically significant postsurgical complication frequencies between groups, with no implants requiring removal or revision.^8^

## Discussion

The aim of this study was to review the current literature on short-term and midterm results of rTSA performed with augmented baseplates and compare with cases using standard baseplates. Overall, clinical outcomes and complication rates were similar in the augmented cohort compared to the standard cohort. There was a higher complication rate amongst the nonaugmented pooled cohort. When comparing bone graft to metal augmentation, similar clinical outcomes were demonstrated; however, bone grafting did demonstrate higher complication rates in 1 study.

Glenoid wear is common and can be difficult to address in the operative setting when performing rTSA. This study builds on a previous study by Ghanta et al who reviewed clinical outcomes of 810 patients who underwent rTSA with metal glenoid augmentation. They found positive clinical and functional outcomes in cases of augmentation and concluded their use is safe and effective in rTSA cases.^3^ Our systematic further summarizes comparative outcomes of augmented vs. nonaugmented rTSA constructs.

Metal augmentation of glenoid base plates is one way to address glenoid bone loss and has had good short-term and midterm outcomes. Glenoid augmentation has also been shown to reduce pain, increase ROM and improve patient reported outcomes and functional scores in those undergoing rTSA. Gulotta, Levin and Park et al demonstrated improvements in active ROM in augmented cohorts compared to standard baseplate rTSA patients. The aforementioned studies also reported similar or better clinical outcomes when comparing augmented to standard glenoid groups including ASES, SST, Constant, and VAS pain scores. Complication rates were significantly less in the augmented group in the study by Park et al, and trended this way in the Levin et al study. Overall reoperation rates were similar between cohorts. These findings are useful and align with the author's predictions regarding glenoid augmentation outcomes in rTSA. Having a well-described and well-studied treatment option to address the difficulties associated with glenoid wear and glenoid bone loss is extremely beneficial. As individuals live longer, glenoid wear may become more prevalent. It would not be surprising to see advancements in augmentation including biomaterial make up to optimize integration, more robust size and shape diversity and customization to individual patient anatomy.

Bone grafting is another method to address glenoid wear. When comparing metal augmentation to bone graft, there were similar overall complication rates, revision rates, ROM, and functional outcome scores found between groups. However, infection, component loosening, and notching were more common in the bone graft group. Nabergoj et al demonstrated significant postoperative improvements in ROM and clinical scores after 2 years in rTSA with BMA and rTSA with BA alone; however the BMA group had greater bone loss and significantly different glenoid morphology (12.5% B1, 12.5% B3, 25% C, 25% D, and 25% E3) than the BA group, which may have lead to a less significant difference in calculated outcomes between groups.

There are several limitations to this study. Patients who received augmented baseplates tended to represent individuals with more glenoid wear. Therefore, if anything, one would expect this to negatively impact the outcomes of the augmented group. We did not observe such a trend in our data. A potential limitation of using augmented baseplates in rTSA is the risk of excessive lateralization and associated soft tissue tensioning, which may increase the likelihood of acromial stress fractures and other postoperative complications. Notably, the included studies are heterogeneous in nature including primary and revision rTSA and variable indications for surgery such as OA and cuff arthropathy. Rehabilitation protocols and surgical technique of various surgeons in this study were not standardized. However, this could allow for greater generalization to the population at large, where there is no single standard of care for the rehabilitation of patients undergoing rTSA. Information on glenoid morphology was not collected but could be the subject of future studies. Additionally, the average follow-up for these patients was just under 4 years. Longer term studies will be needed to determine the longevity of these implants.

## Conclusion

This systematic review demonstrates comparable outcomes with the use of augmented glenoid baseplates to address glenoid bone loss in rTSA when compared to standard baseplates. Complications were equal or less prevalent in the augmented baseplate group, with no difference demonstrated in revision rates. These findings illustrate that augmented baseplates not only can address glenoid bone loss, but provide good clinical and functional outcomes without the risk of increased complication in rTSA.

## Disclaimers

Funding: No funding was disclosed by the authors.

Conflicts of interest: Albert Lin reports a relationship with Arthrex Inc. that includes: consulting or advisory; a relationship with Stryker that includes: consulting or advisory; a relationship with Tornier Inc. that includes: consulting or advisory; a relationship with restor3d Inc. that includes: board membership. All the other authors, their immediate families, and any research foundations with which they are affiliated have not received any financial payments or other benefits from any commercial entity related to the subject of this article.
